# Optimizing Transarterial Chemoembolization in Hepatocellular Carcinoma: Current Strategies, Innovations, and Future Directions

**DOI:** 10.7759/cureus.95128

**Published:** 2025-10-22

**Authors:** Jeraun Dolphin, Juan Zubillaga, Qazi Muhammad Jamal, Afrah Fathima Karimbanakkal Edakkattu, Bhavana Balakrishnan, Aarati Sapkota, Anum Naimat, Aye Nyein, Confidence Obianuju Okorie, Ahmad Mahmood, Aabind S Dev

**Affiliations:** 1 Research, Virscio, New Haven, USA; 2 Gastroenterology and Hepatology, Universidad Central de Venezuela, Caracas, VEN; 3 Internal Medicine, University Hospitals of Leicester NHS Trust, Leicester, GBR; 4 General Medicine, Government Medical College Manjeri, Kerala University of Health Sciences, Manjeri, IND; 5 General Medicine, Kasturba Medical College, Manipal, Manipal, IND; 6 Oncology, Edinburgh Cancer Centre, NHS Lothian, Edinburgh, GBR; 7 Internal Medicine, Fatima Memorial Hospital (FMH) College of Medicine and Dentistry, Lahore, PAK; 8 Acute Medicine, University Hospital Birmingham, Birmingham, GBR; 9 Internal Medicine, Royal Oldham Hospital, Northern Care Alliance NHS Foundation Trust, Oldham, GBR; 10 General Surgery, Xinjiang Medical University, Xinjiang, CHN; 11 Department of Diagnostic and Interventional Radiology, Lakeshore Hospital, Kochi, IND

**Keywords:** chemoembolization, locoregional therapy, postembolization syndrome, radiomics, tace refractoriness

## Abstract

Hepatocellular carcinoma (HCC) remains a major global health challenge with high mortality, particularly in patients with intermediate-stage disease, where curative options are limited. Transarterial chemoembolization (TACE) is the mainstay of treatment in such cases, offering locoregional tumor control by combining targeted chemotherapy and embolization. This review explores current strategies to optimize TACE, including advancements in drug delivery systems such as drug-eluting beads and balloon-occluded techniques, and highlights the importance of patient selection based on Barcelona Clinic Liver Cancer (BCLC) staging, liver function, and performance status. Emerging combination therapies, such as TACE with systemic agents like sorafenib, lenvatinib, and apatinib, or immunotherapies like atezolizumab-bevacizumab, show promise in improving progression-free and overall survival. Additionally, TACE plays a critical role in bridging and downstaging patients for liver transplantation. Future directions focus on integrating radiomics, artificial intelligence, and inflammatory biomarkers to enhance treatment personalization and predict therapeutic response. Despite its established role, TACE is not without risks, including post-embolization syndrome and organ-specific ischemic complications. Continued research is essential to refine selection criteria, minimize adverse effects, and validate innovative approaches, ultimately improving outcomes for HCC patients across diverse clinical scenarios.

## Introduction and background

Hepatocellular carcinoma (HCC) is the most common primary malignancy of the liver and a leading cause of cancer-related mortality worldwide [1]. Despite appropriate treatment, outcomes remain poor [2]. HCC is characterised by a multifactorial and highly complex pathophysiology. As a result, therapeutic options and effective regimens often vary on a case-by-case basis, largely dependent on both clinical factors and a shared decision-making process between the physician and patient [3].

In recent times, the use of combination therapy has been proven to be far superior to the use of monotherapy in the treatment of HCC. Not only does it mitigate the risk of drug resistance, but it also aids in adequate tumour suppression, decreasing drug toxicity, and increasing the overall survival rate [4]. One such treatment modality is transarterial chemoembolization (TACE), a widely used treatment for unresectable localized HCC, which combines both chemotherapy and embolization. Conventional TACE therapy involves intra-arterial injection of chemotherapeutic agents such as mitomycin C along with an embolization agent like ethiodized oil (Lipiodol). This approach induces ischemia while allowing prolonged retention of chemotherapeutic agents within the tumour environment, thereby promoting fibrosis and localized cytotoxicity [5,6]. The success of TACE is highly dependent on appropriate patient selection, as administration in unsuitable candidates may cause harm and reduce overall survival benefits [7]. Additionally, patients must exhibit well-defined nodules, preserved portal flow, and adequate arterial access to facilitate selective embolization [8].

TACE combined with systemic therapy, most notably sorafenib, an angiogenesis inhibitor approved for BCLC stage C HCC, has been explored, though outcomes remain suboptimal in terms of overall patient recovery. Newer agents such as apatinib, a selective vascular endothelial growth factor-2 (VEGFR-2) inhibitor with higher binding affinity than sorafenib, show promise, but their safety and efficacy in combination with TACE remain under research [9].

Locoregional combinations like TACE with radiofrequency ablation (RFA) have shown improved survival in tumors under 7 cm, but the hypoxic environment post-TACE can increase recurrence risks. Similarly, TACE combined with percutaneous ethanol injection demonstrates a synergistic effect, particularly for larger tumors, but requires preserved liver function [10]. Drug-eluting bead TACE (DEB-TACE), which allows controlled and sustained drug release, has shown comparable outcomes to conventional TACE (cTACE). However, no significant difference has been observed in tumor response, time to progression, or overall survival, leaving the choice between DEB-TACE and cTACE to the physician's discretion [11,12]. DEB-TACE is associated with a relatively lower risk of post-procedural pain but a higher risk of hepatic artery and biliary injuries compared to cTACE [13]. Lastly, balloon-occluded TACE (B-TACE), developed in Japan, uses microballoon catheters to enhance drug delivery and has shown superior results in early studies, though the current data is limited by small sample sizes and retrospective designs [14].

Bridging therapy and neoadjuvant use of TACE in patients waiting for liver transplant is the most common use of TACE among other methods, like RFA or microwave ablation. This will help the patient to downstage the tumour in the liver recipient so that the patient can meet the criteria to receive liver transplantation. Additionally, it was found that patients who respond poorly to this type of bridging therapy are at greater risk for pre-transplant tumour-related delisting and post-transplant tumour recurrence [15]. TACE is generally considered safe in most patients; however, a small number of patients may experience complications such as post-embolization syndrome (PES). Ongoing advancements in techniques, along with additional systemic therapies, are enhancing the effectiveness of TACE by improving treatment outcomes, and the key objective of this review is to explore current and future strategies for optimizing TACE [16].

## Review

Background and disease overview

HCC is the most common primary liver cancer and is one of the major causes of cancer-related mortality worldwide, ranked at fourth in mortality [17,18]. Among the deaths caused by cancers in the United States, hepatocellular carcinoma ranks as the ninth leading cause [18]. China accounts for 50% of global HCC patients, and HCC has become the fourth most common cancer and the second leading cause of cancer-related deaths in China [19].

There are multiple risk factors for HCC, with hepatitis B, hepatitis C, hepatitis D, alcohol, non-alcoholic fatty liver disease, obesity, diabetes, and aflatoxins being the common ones. Other risk factors include hereditary hemochromatosis, smoking, glycogen storage disorders, oral contraceptives, and cirrhosis [20].

Hepatitis B infection, alcohol, and hepatitis C infection are the leading causes of HCC globally, and of the mortality associated with this condition. However, different risk factors are attributed as the most common causes of HCC in different geographical regions due to variability in the presence of these risk factors. For instance, about 70% of HCC is attributed to infections by the hepatitis B virus in Asia and Africa. Recently, there has been an increased incidence of HCC attributed to non-alcoholic fatty liver disease in Europe and the United States, and about 40% of HCC has been linked with aflatoxins and p53 mutations in regions such as Africa and China [3].

The underlying pathophysiology in HCC can be broadly categorised into mutations, epigenetic changes, and pathway dysregulations [2]. One of the most common mutations associated with HCC is the mutation of the tumour suppressor p53 gene, as it loses its regulatory functions and its ability to trigger programmed cell death in abnormal cells. Hepatitis B virus integrates its viral DNA into the human genome, which leads to several alteration events, including (1) HBV-promoter induced transcription of host genes, (2) viral-host transcript fusion, which may lead to activation of proto-oncogenes or inactivation of tumour suppressor genes, and (3) disruption of the genome [2]. Hence, HBV increases the risks of cancer by promoting instability of chromosomes and changes to the genome by deletions or translocations. Other mutations commonly found in HCC are CTNNB1 and AXIN1, which are related to the Wnt/β-catenin signalling pathway. Other infrequent but typical mutations related to HCC are PIK3CA and PTEN (PI3K/AKT signalling pathway), KRAS, NRAS, and BRAF (RAS/MAPK signalling pathway), EGFR (growth factor signalling pathway), IDH1 and IDH2 (NADPH metabolism) [2]. Epigenetic mechanisms are inheritable changes in phenotypes caused by how the genes are expressed without affecting the structural integrity of chromosomes or DNA. Epigenetic mechanisms that are related to HCC pathophysiology are (1) changes in methylation, hydroxy-methylation, and acetylation of all or specific DNA regions, (2) modifications of histone proteins that are related to packaging of DNA, and (3) non-coding RNA molecules that control expression of genes [2].

Signalling pathways control interactions between intracellular and extracellular environments and how the cells respond intrinsically to extracellular signals. These pathways control functions ranging from cellular division and growth to differentiation and programmed cell death. Disorders of the Wnt/β signalling pathway, tyrosine kinase receptor signalling pathway, vascular endothelial growth factor (VEGF) pathway, JAK/STAT pathway, and transforming growth factor-beta pathway (TGF-β) are commonly reported in relation to HCC pathophysiology [2].

Serum markers like alpha fetoprotein (AFP), AFP-L3, and DCP are used alongside imaging modalities for HCC screening, with AFP showing limited sensitivity and possible false elevations in non-HCC conditions. While ultrasound is commonly used, CT and MRI offer higher sensitivity for small tumours, especially when combined with serum markers for improved diagnostic accuracy [18].

The Liver Imaging Reporting and Data System (Li-RADS) is used in high-risk patients with liver cirrhosis and chronic HBV patients for classifying liver lesions on CT or MRI based on certain characteristics such as arterial phase hyperenhancement, non-peripheral washout, size, capsule, and threshold growth; an increase in size of 50% or more within six months' time. The lesions are classified into definitely benign (LR-1), probably benign (LR-2), probably or definitely malignant but not HCC (LR-M), intermediate probability of malignancy (LR-3), probably HCC (LR-4), and definitely HCC (LR-5). If the tumour is in the vein, then it is categorised as tumour in vein (LR-TIV), and if the tumour cannot be categorised due to image degradation or omission, then it is categorised as LR-NC [21]. Li-RADS has the highest sensitivity and accuracy amongst non-invasive diagnostic criteria for diagnosis of HCC when compared with American Association for the Study of Liver Diseases (AASLD) criteria, Korean Liver Cancer Study Group and the National Cancer Center, Korea (KLCSG-NCC), European Association for the Study of the Liver and the European Organization for Research and Treatment of Cancer (EASL-EORTC), and Organ Procurement and Transplantation Network (OPTN) and the United Network for Organ Sharing (OPTN-UNOS) criteria [22].

As HCC is more common in chronically inflamed and cirrhotic liver secondary to various aetiologies, different staging systems have been used to predict prognosis in HCC, including the Child-Pugh score, the Albumin-Bilirubin (ALBI) score, and the Model for End-Stage Liver Disease (MELD). The ALBI score has been reported to be more accurate in predicting prognosis in HCC as compared to the Child-Pugh and MELD scores. The ALBI score has also been used to predict tumour relapses and liver failure in patients after hepatectomy for HCC [23].

The Barcelona Clinic Liver Cancer (BCLC) staging system is widely accepted and used to classify HCC into different stages based on tumour burden, liver function, and performance status. It classifies HCC into stages of very early stage (0), early stage (A), intermediate stage (B), advanced stage (C), and terminal stage (D). BCLC not only classifies but also advises on treatment options for different stages of HCC [24]. However, in practice, treatment given in different stages of HCC deviates from the BCLC guidance, especially for stages B and C, and performance status, being a subjective measure, seems to be influential in deviating from the BCLC algorithm [25]. The BCLC staging system is an evidence-based, comprehensive staging system that is simple to follow and not only guides treatment options but also predicts prognosis in terms of overall survival. On the other hand, it has some limitations as it doesn’t take into account molecular markers (AFP, AFP-L3, PIVKA-II), there is no elaboration on intra-hepatic or extra-hepatic vascular or biliary invasion, no detailed utilization of liver function parameters, and relies on performance status with no consideration of comorbidities [26].

Role and rationale of TACE in HCC management

Mechanism of Action: Dual Role of Embolization and Chemotherapy

TACE is a cornerstone of the treatment of HCC, especially for patients not fit for curative surgery or transplant. It involves identifying the blood supply of liver tumours, administering focused chemotherapy, and blocking the blood supply at the same time [27,28]. 

Chemotherapy effect: In HCC, the tumour is primarily vascularized by the hepatic artery, while the portal venous circulation predominantly supplies the surrounding liver parenchyma. This anatomic imbalance is the basis for TACE, which permits selective chemotherapy infusion into the arteries that feed the tumours [11,27].

Vascular embolization: After the chemotherapy infusion, embolic agents are administered in order to embolize the feeding arteries. Gelatin sponge particles, polyvinyl alcohol (PVA) particles, and DEB are some of the most widely used embolic agents. Injecting these agents results in ischemia and enhances chemotherapy-induced toxicity. Localized chemotherapy combined with arterial embolization shows a synergistic action, promoting destruction of the tumour to a certain degree, while not affecting normal liver parenchyma [11,27]. 

The dual action of TACE (chemotherapy and embolization) provides a valuable advantage in intermediate-stage HCC. Embolization-induced ischemia augments chemotherapy-induced cytotoxicity and tumour killing. Thus, it ablates the tumour and demonstrates durable efficacy. Local chemotherapy also provides lower systemic exposure, leading to fewer side effects [11,27].

Conventional vs. DEB-TACE

In cTACE, an oily emulsion (e.g., Lipidol) with a chemotherapeutic drug is delivered by transcatheter injection, followed by embolization of the blood supply using particles of gelatin sponge or polyvinyl alcohol (PVA) to induce vessel occlusion and death of the tumour cell. Despite this, lipidol and the chemotherapeutic agents are not bound chemically, leading to uncontrolled release of drugs and possible systemic toxicity. It may result in post-embolization syndrome (PES) and other complications such as ischemic pain and liver failure [28,29].

DEB-TACE uses microspherical beads preloaded with chemotherapeutics. These beads permit a slow and steady release of chemotherapy agents over a few days, resulting in higher local concentrations with the greatest antineoplastic effect and less systemic toxicity [29].

Several randomized controlled trials (RCTS) and meta-analyses have compared the efficacy and safety of cTACE and DEB-TACE. Complications after treatment, including abdominal pain and bile duct dilation, were observed more frequently in DEB-TACE patients [28,30]. Fan et al. stated the median overall survival (OS) of the patients in DEB-TACE group (11.4 months, 95% confidence interval [CI]: 10.1−14.0) was better than that in cTACEgroups (9.1 months, 95% CI: 9.6−12.3) (hazard ratio (HR) = 2.46, 95% CI: 1.50−4.04, p < 0.001) [31].

In a meta-analysis by Zou et al., DEB-TACE achieved the complete response rate [odds ratio (OR) 1.38, 95% CI 1.01-1.89], overall survival rate (OR 1.41, 95% CI 1.01-1.98). It is associated with reduced systemic toxicity and lower post-embolization syndrome incidence [29,31].

However, the two modalities' overall survival and tumour response rates remain comparable. The difference between TACE and DEB-TACE is still debated. Thus, it should be individualized based on the patient's liver function, performance status, clinical profile, and tumour characteristics [11].

Indications based on the Barcelona Clinic Liver Cancer staging (BCLC) 

The BCLC staging is essential for the stratification of HCC patients and for making decisions regarding treatment [27,32]. TACE is the standard treatment for BCLC Stage B (intermediate stage). Tumours are limited to liver parenchyma with no signs of vascular invasion or metastasis, preserved liver function (Child-Pugh A/B), and patients with a performance status of 0-1 (ECOG) [27,33]. Surgery, ablation, or transplantation is the treatment of choice for early-stage BCLC stage A. However, if these treatments are contraindicated, TACE is an alternative treatment. Patients with stage A HCC who meet the Milan or University of California, San Francisco (UCSF) criteria are candidates for transplantation. TACE can also serve as a bridge therapy to transplantation or tumour downstaging [11,32].

For those with advanced-stage (C) HCC with vascular invasion or extrahepatic spread, systemic treatment with tyrosine kinase inhibitors (TKI) such as sorafenib or lenvatinib is recommended as the first-line treatment. These agents share a common mechanism of inhibiting VEGF receptors, thereby suppressing tumour angiogenesis, while also targeting other tyrosine kinase receptors to interrupt tumour cell signaling pathways. If TACE is implemented in combination with systemic therapy, it delays tumour progression, provides better efficacy, and improves overall survival. As per Silk et al., median overall survival (OS) was 19.2 months with the combination therapy and 13.4 months with sorafenib (HR, 0.66 (95% CI: 0.52-0.85); P = 0.0009). However, TACE is contraindicated in complete portal vein thrombosis, as the risk of hepatic ischemia and secondary liver failure is too high [32,34]. 

Importance of patient selection

Patient selection plays a vital role in ensuring optimal outcomes and minimizing complications from TACE. One key consideration is the Eastern Cooperative Oncology Group (ECOG) performance status, where patients with a score of 0 to 1-indicating full activity and in self-care ability-are suitable candidates, while those with a score above 2 are more prone to treatment intolerance and postprocedural morbidity [35]. The ALBI score provides a more objective assessment of liver function than the traditional Child-Pugh score; patients with ALBI grades 1 or 2 are best suited for TACE, whereas those with grade 3 face a significantly increased risk of hepatic decompensation [36,37]. Portal vein patency is another crucial criterion, typically assessed using contrast-enhanced CT or MRI. Complete portal vein thrombosis raises the risk of ischemic liver injury and potential liver failure, although selected patients with segmental or partial thrombosis and adequate collateral circulation may still benefit from TACE [38]. Additionally, tumour burden - evaluated through factors such as tumour size and number using tools like the “up-to-seven” criteria - affects eligibility, with those exceeding the threshold at higher risk of complications and demonstrating lower treatment response rates [32].

Combination therapies

TACE, while being the standard of care for intermediate-stage HCC, is not without its limitations. TACE has been shown to increase tumour hypoxia, resulting in the upregulation of the hypoxia inducible factor-1α (HIF-1α) [39,40]. Increasing expression of HIF-1α sequentially results in an upregulation of proangiogenic factors such as VEGF and platelet-derived growth factor (PDGF), promoting tumour angiogenesis [39,40]. TACE has been tested in combination with several antiangiogenetic agents to limit the increase of these proangiogenic factors to improve survival outcomes.

TACE With Sorafenib

TKIs such as sorafenib function by inhibiting the downstream signalling pathways that play a vital role in angiogenesis, hence the rationale for their use with TACE [40]. Chang et al. reported that both the SPACE and TACE 2 trials compared TACE with sorafenib and TACE alone; however, these trials failed to show any clinical advantage of combining sorafenib with TACE [13]. The SPACE trial reported that median overall survival (OS) was not reached, whereas the TACE2 trial reported a median progression-free survival (PFS) of 238 days vs 235 days in the control group (p=0.94) [41,42]. Kudo et al. suggested that the Response Evaluation Criteria in Solid Tumor (RECIST) version 1.1 and the modified RECIST (mRECIST) criteria employed in the TACE 2 and SPACE trials, respectively, may not have been suitable for capturing the progression of intermediate-stage HCC, as they are not indicative of TACE failure [39]. Using untreatable (unTACEable) progression/TACE failure as the criteria for progression, Kudo et al. demonstrated in the TACTICS trial that TACE with sorafenib was more clinically advantageous than TACE alone (median PFS of 25.2 months vs 13.5 months, respectively) in unresectable HCC (no BCLC stage was indicated) [39]. Kudo et al. suggested several other reasons for the more positive outcome, one being the longer median duration of sorafenib treatment versus what was reported in the SPACE and TACE 2 trials (38.7, 21, and 17.1 weeks, respectively), suggesting the SPACE and TACE 2 were also too short in duration for a proper endpoint measurement [13,39,41,42]. In 2022, Kudo et al. published the final results of the TACTICS trial, reporting an updated PFS similar to what was reported initially (22.8 compared to 25.2 months initially) [39,43]. OS, which was not reported in the initial publication, showed no advantage of TACE with sorafenib over TACE alone (36.2 months versus 30.8 months, respectively), indicating that while TACE plus sorafenib may increase PFS, OS was relatively unaffected [39,43].

In the TACTICS-L trial, Kudo et al. suggested that the lack of OS benefit seen in the TACTICS trial could be a result of the considerable amount of subsequent treatment in the TACE alone group, extending post-progression survival [44]. In a retrospective study, Liu et al. reported similar results as the TACTICS trials in patients with advanced-stage HCC; however, OS was significantly increased in patients treated with TACE and sorafenib combination in this case (22.9 months in the combination group vs 12.1 months in the control group) [45]. This indicates that the subsequent treatment in the TACE alone group, reported by Kudo et al., may have played a role in the lack of OS benefit, as they suggested [44,45]. It is also important to note that TACE-sorafenib patients experienced higher incidences of all grades of adverse events (AEs) than TACE only [39,43].

TACE With Lenvatinib

Lenvatinib is a new first-line drug for the treatment of liver cancer and is a more potent VEGF inhibitor than sorafenib [44,46]. The TACTICS-L trial utilized the same progression criteria as the TACTICS trial to evaluate the efficacy of TACE with lenvatinib in patients with unresectable intermediate-stage HCC; however, a TACE-alone treatment was not included in the experimental design [39,44]. The median PFS was reported to be 28 months at the additional follow-up [44], which was higher than what was reported in the TACTICS trials for TACE with sorafenib [39,43]. The reported complete response (CR) and the objective response rate (ORR) were also higher in the TACTICS-L trial than in the TACTICS trial (67.7% and 88.7% in the TACTICS-L trial versus 28% and 57% in the TACTICS trial), whereas OS was not reached [39,44]. Although statistical significance was not determined, these results support the efficacy of TACE combined with lenvatinib, suggesting that this combination may be more clinically effective than TACE with sorafenib (TACTICS-L Trial was carried out in BCLC Stage B unresectable HCC). Xu et al. also provided evidence of TACE with lenvatinib being clinically superior to TACE with sorafenib in patients with intermediate-advanced HCC (10 vs 6.5 months median PFS and 13 vs 8 months median OS, p<0.01 for both) [46]. These results showcase the strong potential of TACE-lenvatinib combination therapy and its advantage over TACE-sorafenib. No new safety concerns had been identified in the TACTICS-L trial [44]; however, Xu et al. reported an increase in select AEs such as Hand-foot syndrome (p=0.004), diarrhoea, hypertension, and rashes; however, no differences were observed in other AEs [46].

TACE With Apatinib

Similar to sorafenib and lenvatinib, apatinib is another small-molecule drug with antiangiogenic properties, independently developed by Chinese scholars [47]. When combined with TACE, a significant increase in the median PFS was observed compared to TACE alone (20 vs 14 months, p<0.05) in intermediate-advanced HCC patients; however, no significant difference was observed in the OS for both groups (35 vs 28 months, p=0.06) [48]. The authors suggested that the patients may have received other treatments at baseline and/or patients may have taken other treatments during disease progression, possibly affecting OS; however, these variables were not accounted for in the outcome calculations [48]. In their systematic review and meta-analysis of 23 eligible studies, Zhao et al. reported a significantly better half and one-year survival in intermediate-advanced HCC patients who received TACE-apatinib compared to patients who received TACE alone (Pooled odds ratios (OR)=2.741 and 2.284, respectively), indicating that TACE-apatinib does, in fact, increase patient survival compared to TACE alone [49]. When TACE plus Apatinib was retrospectively compared to TACE plus sorafenib in patients with unresectable HCC, TACE-sorafenib showed better efficacy than TACE-apatinib (16.79 vs 14.76 months, p=0.049 for PFS and 20.66 vs 17.69, p=0.013 for OS) [47]. Unfortunately, TACE-apatinib treatment resulted in higher AEs compared to those who received TACE-sorafenib before dose adjustment; however, no difference was observed after dose adjustment [47]. Gong and Li also reported higher incidences of AEs in TACE-apatinib compared to TACE alone [9]. While TACE-apatinib combination therapy is shown to be much more effective than TACE alone, the evidence also highlights its potential disadvantages compared to TACE-sorafenib, potentially making it the less preferred option of the two combinations; however, more data is needed to validate these findings. 

TACE With Atezolizumab + Bevacizumab

Atezolizumab + bevacizumab combination therapy was approved in 2020, after strong evidence of a clinically meaningful increase in median OS (25.8 vs 21.9 months, respectively) and PFS (12.6 vs 8.6 months, respectively) compared to sorafenib was reported by the IMbrave150 trial, making it one of the newer options of multiple systemic treatments available for HCC, as well as one of the first-line treatments [50,51]. Atezolizumab, an anti-programmed death-ligand 1 monoclonal antibody, and bevacizumab, an anti-VEGF monoclonal antibody, were approved in over 85 countries for the treatment of patients with unresectable HCC [51], supporting the rationale for their combination with TACE to observe their synergistic effect. Wang et al. reported that in patients with intermediate-stage HCC beyond the up-to-seven criteria, the median PFS and OS were not reached when TACE was combined with atezolizumab and bevacizumab; however, the median follow-up duration was only 11.7 months, which may not have been long enough to properly assess these outcomes [52]. However, the best ORR and disease control rate (DCR) according to the RECIST 1.1 and mRECIST criteria were reported at 42.9% and 100% versus 61.9% and 100% respectively, while the most frequent treatment-related AEs (TRAEs) at all levels were fever, with hypertension being the most common grade 3/4 TRAE [52]. Initial results show that TACE in combination with atezolizumab + bevacizumab is very promising; however, the current lack of available data highlights the need for more studies to assess the anti-tumor synergy of this combination in comparison to TACE alone and/or other combination treatments with appropriate follow-up durations to properly assess study outcomes.

TACE Combination With Ablation Modalities

Percutaneous ablation has become a vital alternative HCC treatment following the development of ultrasound guidance and other imaging techniques [17]. Many of these ablation methods have been approved, including radiofrequency ablation (RFA), microwave ablation (MWA), and percutaneous ethanol injection (PEI) [17], which we will cover in this review. 

TACE with RFA: A retrospective analysis done on patients with tumour sizes ranging from 3 to 10 cm showed that TACE + RFA increased PFS (9.13 vs 4 months, respectively) and OS (27.57 vs 12 months, respectively) significantly compared to TACE alone (p<0.001 for both) [53]. Major complications were reported in only 4.8% (6/124 patients) of the TACE + RFA group and were resolved with appropriate medications, indicating the relative safety of the combination treatment [53]. The efficacy of TACE + RFA was also seen in another retrospective study in patients with HCC tumours ≤ 5cm [54]. While no other treatment groups were included, 1-, 3-, 5,- and 7-year OS rates were 100%, 95.2%, 95.2% and 95.2% respectively, with only 23.7% of patients experiencing minor Grade B complications with no major complications [54]. Compared to RFA only, TACE + RFA showed significantly higher 5- and 7-year OS rates than RFA only (p=0.001) [55]. These results demonstrate the superiority of TACE + RFA over singular TACE and RFA monotherapies, aligning with current clinical evidence and supporting the widespread use of this treatment combination [17].

TACE with MWA: In a systematic review and meta-analysis, Wang et al. reported that across the nine studies they looked at, in patients with unresectable tumors or unwillingness of resection, the 1-, 2-, and 3-year survival rates were in favor of TACE + MWA groups as opposed to TACE alone groups with pooled odds ratios (OR) of 3.29, 2.82, and 4.50, respectively [56]. No difference in severe AE was observed between both groups in four of the included studies (p>0.05), providing evidence for not only the strong clinical efficacy of TACE + MWA but also its safety [56].

TACE with PEI: A meta-analysis published in 2015 reported that across 19 RCTs, TACE + PEI combination significantly improved the 1-, 2-, and 3-year survival rate (pooled risk ratios (RR) of 1.25, 1.64, and 2.27, respectively) compared to TACE or PEI alone [57]. The efficacy of the TACE + PEI combination was also seen in a study published in 2025 [10]. Feng et al. analysed the complete ablation rate of target lesions with diameters less than or equal to 3 cm at the two-month follow-up in 62 patients [10]. Although the study compared guidance modalities for PEI, complete ablation was achieved in 54 of 67 total lesions (80.6%) with no severe complications being reported during treatment or follow-up [10]. It was reported that an increase in lesion diameter was associated with a reduced likelihood of complete ablation [10]. Nevertheless, these results highlight the strong anti-tumour synergy of the TACE + PEI combination, resulting in its high clinical efficacy. 

TACE as a Bridge to Liver Transplantation 

Liver transplantation, the definitive treatment for HCC, is often associated with a prolonged waiting period. During this interval, TACE plays a pivotal role in bridging (maintaining transplant eligibility of tumours that initially meet eligibility criteria) and downstaging (bringing tumours that did not previously meet criteria within transplant eligibility) [58,59]. This was emphasised by major hepatology guidelines like the EASL (European Association for the Study of Liver) clinical practice guidelines (2018) [60] and the AASLD (American Association for the Study of Liver Diseases) guidelines (2018 update) [59]. Considering recent evidence highlighting TACE as an efficient bridging and downstaging modality, a consensus report was released by the European Society of Organ Transplantation (ESOT) in 2023 [61]. Reinforcing the bridging efficacy of TACE is the recent evidence provided by a study conducted by Minici et al. [62]. It showed that patients with early HCC, subjected to TACE with degradable starch microspheres (DSM-TACE), maintained their transplant eligibility at a rate of 96% for six months and 92% for 12 months [62]. A systematic review by Parikh et al. (2015) showed a 48% success rate in the downstaging of HCC to within Milan criteria, with acceptable post-transplant recurrence (16%) and five-year survival rates [63]. Augmenting these findings is a recent multicenter study by Tabrizian et al. [64], wherein patients successfully downstaged with TACE had 10-year survival outcomes (52.1%), which was comparable to that of those who already met Milan criteria at diagnosis (61.5%) [64].

The efficacy of TACE in this context was further supported by a retrospective analysis of 580 HCC patients undergoing TACE using DEB-TACE, conducted by Lim et al. [65]. Most of them were successfully bridged (97.1%), and over half of them (58.4%) were downstage. Patients treated with smaller DEB (100-300 μm) had significantly higher response rates, with 99.5% bridged and 63.8% downstage, emphasising the importance of particle size in treatment success rates [65].

Together, these studies establish TACE as a reliable modality for both bridging and downstaging in HCC patients, emphasising its importance in the management of HCC prior to liver transplantation. However, there are different modalities of TACE, such as conventional TACE (cTACE), DEB-TACE, DSM-TACE, and others, each with its own degree of efficiency. This is a limitation, as not all studies do not evaluate these modalities equally, and hence the findings mentioned above cannot be generalised.

Impact on Transplant Eligibility 

The Milan criteria, first described by Mazzaferro et al. [66], which define liver transplantation eligibility based on tumour size and number (a single tumour ≤ 5 cm or up to 3 tumours each ≤ 3 cm), remain the gold standard for liver transplantation eligibility. However, due to their restrictive nature, other criteria have been introduced. The University of California, San Francisco (UCSF) criteria (which allow slightly larger tumours) have been seen to give comparable outcomes to those of the Milan criteria as per a systematic review and meta-analysis conducted by Bento de Sousa et al. [67]. Further expanding the transplant eligibility is the up-to-seven criteria (where the sum of the largest tumour size in centimetres and the number of tumours is ≤ 7), which gave acceptable survival rates according to a retrospective analysis conducted by León Diáz et al. [68]. Additionally, the United Network for Organ Sharing (UNOS) criteria (based on Milan but including additional elements like AFP levels and treatment response) have also been introduced. A systematic review and meta-analysis by Tan et al. [69] demonstrated favourable outcomes in patients who were downstaged to UNOS criteria, supporting its inclusion as an expanded eligibility criterion. Multiple studies have assessed the effectiveness of TACE in both bridging and downstaging tumours to meet these criteria, some of which have been discussed below.

Yu et al. (2016) studied 60 HCC patients who were undergoing TACE, of whom 17 patients did not initially fit into the UCSF criteria. The data obtained provide further evidence of TACE’s efficacy. A total of 73.9% were successfully downstaged, and 40% of the total cohort underwent successful liver transplantation. Post-transplantation recurrence was observed only in 12.5% of cases [70].

Yao et al. corroborated this in a comparative evaluation of 118 successfully dowstaged patients and 488 patients who met Milan criteria at diagnosis. The five-year post-transplant survival rates were 77.8% and 81% respectively, showing no major difference [71]. These findings further substantiate the efficacy of downstaging by TACE, as also mentioned in the EASL and AASLD guidelines [72]. 

In another study, Orlacchio et al. (2015) showed that TACE using degradable starch microspheres (DSM-TACE) successfully downstaged patients who were initially beyond the up-to-seven criteria, ensuring their eligibility for liver transplantation [72].

**Figure 1:**
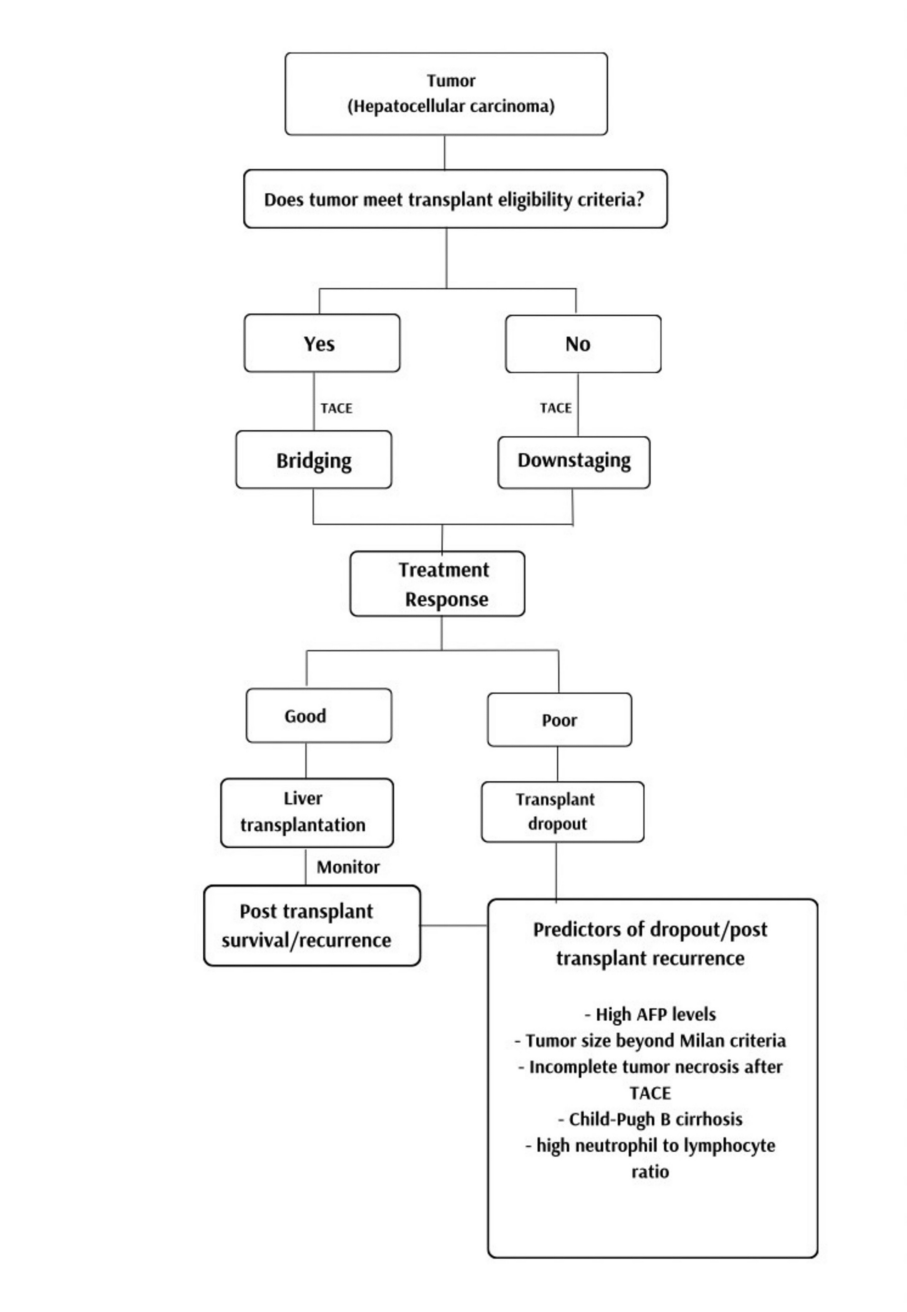
Role of TACE in bridging/downstaging in hepatocellular carcinoma TACE: transarterial chemoembolization; AFP: alpha fetoprotein Image Credits: Created by author Afrah FK Edakkattu.

These studies cumulatively suggest that TACE is an efficient modality for bridging and downstaging hepatocellular carcinoma patients, expanding the cohort eligible for liver transplantation and improving post-transplant outcomes (Figure 1). 

Predictors of transplant dropout and recurrence

Despite the utility of TACE, a subgroup of patients undergoing this treatment modality still face transplant dropout and post-transplant recurrence due to several factors. According to a study conducted by Benkö et al. (2022), although TACE and radioembolization were effective bridging therapies, transplant dropout was linked to high baseline AFP levels, larger tumour size beyond Milan criteria, and incomplete tumour necrosis [73]. In addition to this, Mehta et al. showed that HCC patients who had low AFP levels (< 500 ng/mL) after locoregional therapies like TACE were associated with lower transplant dropouts and post-transplant recurrence [74].

Corroborating this is a retrospective cohort study conducted by Yao et al. (2015), wherein it was found that high baseline AFP levels (≥ 1000 ng/mL) and Child Pugh B cirrhosis are significant predictors of transplant dropout in patients who were downstaged with TACE for hepatocellular carcinoma [71].

Recurrence following liver transplantation remains a major concern. Jeong et al. (2021) conducted a retrospective study among 51 HCC patients who received TACE, and observed that microvascular invasion was linked to post-transplantation recurrence [75]. Later, in 2024, Lazzarotto-da-Silva et al. showed that incomplete response to TACE is associated with microvascular invasion, which, as mentioned earlier, is an indicator of post-transplantation recurrence [76]. Substantiating this, Yin et al. (2022) showed that incomplete tumour response to TACE is related to higher post-transplantation recurrence and lower survival rates [15]. Therefore, a favourable response to TACE may be an indirect predictor of a lower risk of recurrence following liver transplantation. 

Tabrizian et al.’s multicenter cohort study (2022) identified key predictors of HCC recurrence and poor survival post-transplant, including elevated AFP levels (≥ 20 ng/mL), poor tumour necrosis as evidenced by large viable tumour size on explant pathology, and a high neutrophil to lymphocyte ratio (NLR >5) at the time of transplantation [64].

Thus, as mentioned earlier, even though TACE is useful in bridging and downstaging HCC tumours such that they fit into liver transplant eligibility criteria, it has its challenges of transplant waitlist dropout and post-transplant recurrence. These can be minimised with careful patient selection and monitoring of therapy, thereby obtaining favourable treatment outcomes (Table 1).

**Table 1:** Predictors of transplant dropout and post-transplant recurrence AFP: alpha fetoprotein

Predictor	Reference study	Significance
High baseline AFP levels	Yao et al. (2015) [71], Mehta et al. (2019) [74], Benkö et al. (2022) [73], Tabrizian et al. (2022) [64]	High transplant dropout, high post-transplant recurrence, low post-transplant survival rates
Tumor size beyond Milan	Benkö et al. (2022) [73]	High post-transplant dropout
Incomplete tumor necrosis	Benkö et al. (2022) [73], Tabrizian et al. (2022) [64], Yin et al. (2023) [47]	High transplant dropout, high post-transplant recurrence, low post-transplant survival rates
Child-Pugh B cirrhosis	Yao et al. (2015) [71]	Post-transplant dropout
Microvascular invasion	Jeong et al. (2021) [75]	Incomplete tumour necrosis, high post-transplant recurrence
Neutrophil to lymphocyte ratio >5	Tabrizian et al. (2022) [64]	High post-transplant recurrence, low post-transplant survival rates

Adverse events and safety profile 

TACE is generally considered a safe treatment, yet it carries a range of adverse effects. The most common is post-embolization syndrome (PES), occurring in up to 47% of patients, characterized by fever, abdominal pain, and gastrointestinal symptoms. Its severity is linked to inflammatory cytokine release, with low skeletal muscle index being a contributing factor due to its role in immune regulation [77]. Ischemic complications-such as pancreatitis 0.9-2%, biliary complications 0.5-10%, and liver abscesses 0.3-1.3%-may result from non-target embolization due to the hepatic artery's extensive branching [78]. Liver dysfunction, leukopenia, and skin reactions 2-24.5% have been reported post-TACE, though severe adverse events appear less frequent in combination therapies compared to TACE alone [34, 79].

Acute kidney injury (AKI) is another serious complication, especially in diabetic or elderly patients, with TACE-related AKI increasing short-term mortality risk by nearly fivefold [48]. Sorafenib, commonly used in combination with TACE, is associated with dermatological and gastrointestinal side effects but does not significantly increase TACE-related complications [55]. Rare but severe complications include spinal cord ischemia, HCC rupture (with up to 75% mortality), and diaphragmatic injury-particularly when TACE is performed via the inferior phrenic artery [19]. Gastrointestinal bleeding may also occur due to non-target embolization or vascular anatomical variations. Despite these risks, understanding patient-specific factors can help anticipate and mitigate many of these adverse outcomes.

Management strategies and prevention

Several preventive and management strategies have been proposed to minimize complications following TACE. The prophylactic administration of corticosteroids and 5-HT3 receptor antagonists has been shown to reduce the incidence of post-embolization syndrome (PES), with lipoidal and dexamethasone emulsions demonstrating significant efficacy in lowering its occurrence [28]. Skin necrosis and related injuries may be prevented by prophylactic embolization of cutaneous branches or through the topical application of ice, which induces vasoconstriction [80]. In cases where high-grade fever persists for over two weeks post-procedure-particularly in high-risk patients-liver abscess should be suspected, warranting imaging evaluation. Management primarily involves percutaneous aspiration, while prophylactic antibiotics administered before and after TACE can further reduce this risk [80].

Biliary complications such as biloma and fibrotic strictures, resulting from ischemic necrosis of bile ducts, can be managed with endoscopic retrograde cholangiopancreatography (ERCP) or percutaneous drainage in cases of infection [80]. Preventing gastrointestinal (GI) bleeding and ulcers requires careful angiographic assessment, with coil embolization being the standard preventive measure. The use of anti-reflux microcatheters or micro-balloon catheters has also proven beneficial. Prophylactic H₂ receptor blockers or proton pump inhibitors (PPIs) are often prescribed pre-procedure [81]. Chemotherapy-induced GI ulcers may resist standard medical therapy, necessitating endoscopic evaluation to exclude variceal bleeding or other causes [80]. Diaphragmatic injury can be minimized by selectively embolizing tumor feeders instead of the entire inferior phrenic artery [80]. In cases of HCC rupture, contrast-enhanced CT helps identify the bleeding source; TACE can achieve rapid hemostasis in hemodynamically unstable patients, while conservative treatment is preferred for stable cases. Additionally, embolization followed by staged hepatectomy has been associated with improved survival outcomes [82,83].

Imaging and biomarker-based monitoring

Radiological Assessment

Radiomics has shown promising value in predicting HCC characteristics such as tumour grade, microvascular invasion (MVI), treatment response, and prognosis [84]. Modified Response Evaluation Criteria (mRECIST) is a tumour response evaluation criterion specifically adapted for HCC. It uses unidimensional measurements of enhancing tumour areas to define response as complete response (CR), partial response (PR), stable disease (SD), progressive disease (PD) [85]. 

A CR is defined as the disappearance of all intratumoral arterial enhancement in target lesions. A PR requires at least a 30% reduction in the sum of diameters of viable (enhancement in the arterial phase) target lesions. SD is when the tumour changes do not meet the criteria for either partial response or progressive disease. PD is indicated by a 20% or greater increase in the sum of the diameters of viable target lesions [86].

Magnetic resonance imaging (MRI) is the preferred method for evaluating the effects of TACE in HCC patients [84]. In a study by Zhou et al., FS-T2WI and arterial-phase (phase A) and portal venous-phase (phase P) dynamic contrast-enhanced MR images of HCC patients before TACE were retrospectively analysed, radiomic features were extracted and screened, and clinical data, such as inflammatory indicators, were assessed. To analyse the value of combining multisequence MRI radiomic features and inflammatory indicators, a multisequence radiomic-clinical joint prediction model was constructed [84].

Liu et al. studied 140 HCC patients treated with TACE using a machine learning model integrating radiomic features, BCLC staging, and the ALBI score. Their logistic regression model predicted tumour response with AUCs of 0.813 (training) and 0.781 (testing). Survival analysis showed that predicted response, ALBI, satellite nodules, and BCLC stage were independent predictors of overall survival [86]. These findings suggest that radiomics, combined with clinical data, enhances the prediction of treatment response and prognosis in HCC patients receiving TACE, supporting its potential in guiding personalised treatment strategies [86].

Inflammatory Markers

Inflammatory markers like NLR and platelet lymphocyte ratio (PLR) are the most ensuring biomarkers for surveillance of HCC as they can be easily measured [13]. NLR indicates systemic inflammation and is associated with cancer progression and metastasis. High neutrophils suggest tumour cell proliferation, tumour angiogenesis, metastasis, and disruption of the acquired immune system, whereas low lymphocyte counts indicate impaired anti-tumour immune responses, thus enabling tumour progression and metastasis [13]. The other blood markers, such as PLR, aspartate aminotransferase-to-alanine aminotransferase ratio, or C-reactive protein to albumin ratio combined with NLR, can be used as a prognostic marker. But because of different cutoff values of NLR, different study limits the clinical use and cause confusion. Studies including large cohorts of patients are required to establish the most appropriate NLR cutoff value, which provides good sensitivity and specificity [13]. 

Studies show that high NLR and PLR are linked to poor outcomes in HCC patients undergoing TACE. NLR >3 predicts early disease progression and poor tumour control. While radiomic features and inflammatory markers have been studied separately, to our knowledge, Zhou et al. is the first to combine MRI radiomics with NLR and PLR to predict prognosis. The limitations include the retrospective design and small sample size [13].

Prognostic Scores

Multiple scoring systems help guide TACE treatment in HCC patients. Hepatoma arterial-embolisation (HAP) score displayed an overall better discriminatory ability in predicting OS with a c-score of 0.68 (95%CI 0.64-0.70) compared to the Assessment for Retreatment with TACE (ART) score (c-score 0.57, 95%CI 0.53- 0.60). Therefore, the HAP score has better predictive accuracy for overall survival than the ART score and is useful for initial TACE candidate selection [87]. Several traditional scoring systems, like Child-Pugh, STAT, mSNACOR, and BCLC, have been used to predict the outcome of TACE in patients with HCC. They are used depending on the treatment goal (palliative vs. transplant) [88].

Two scoring systems, the ART and ABCR (AFP, BCLC, Child-Pugh Response), have been developed to help guide retreatment decisions. The ART score considers AST increase, worsening Child-Pugh score, and lack of radiologic response, with a score >= 2.5 suggesting limited benefit from further TACE. The ABCR score includes AFP >= 200 ng/mL, BCLC stage, Child-Pugh increase >=2, and radiologic response, with a score >=4 indicating poor outcomes [88]. However, both scores have limited validation and are not incorporated into official guidelines. Therefore, clinicians should consider other factors like overall liver function, tumour progression, and patient condition rather than just solely relying on this score when deciding whether to continue TACE [88].

Recently, newer and simpler models such as ALBI-TAE and the six-and-twelve score have shown better performance. The Pre- and Post-TACE-Predict models, which incorporate mRECIST response and routine clinical features, allow individualised risk stratification and help guide TACE decisions more effectively [88] (Table 2).

**Table 2:** Summary of prognostic scores HAP: Hepatoma Arterial-Embolisation Prognostic score; AFP: alpha fetoprotein; TACE: transarterial chemoembolization; ART: Assessment for Retreatment with TACE; ABCR: AFP, BCLC, Child-Pugh Response; STATE: Selection for TACE; mSNACOR: Modified Selection for TACE Treatment and Retreatment; BCLC: Barcelona Clinic Liver Cancer Staging System; ECOG PS: Eastern Cooperative Oncology Group Performance Status

Score	Variables	Prognostic stratification	Clinical utility
HAP	AFP >400 ng/dL; serum albumin <35g/L; total bilirubin >17 mmol/L; tumor diameter >7 cm	HAP A = 0 points, HAP B = 1 point, HAP C = 2 points, HAP D >= 3 points	Screening tool prior to initial TACE; identifies suitable candidates for TACE.
ART	Child-Pugh increases following TACE; radiologic tumor response; AST >25% from baseline	Low risk < 2.5 points, high risk >= 2.5 points	Early detection of chemoembolization failure.
ABCR	AFP >= 200 ng/ml; BCLC stage; Child-Pugh score >=2; radiologic response >=4	0-1 points = likely benefit, >= 2 points = poor outcome	Decides to repeat TACE vs stop and consider systemic therapy
Pre-TACE predictive models	Tumor burden (size, number); AFP -ALBI grade; ECOG PS	Stratifies into risk groups (low/medium/high)	Predicts initial TACE outcomes
Post-TACE Predict	Radiological tumor response (mRECIST); AFP	Stratifies into risk groups (favourable/unfavourable)	Predicts prognosis after first TACE; retreatment decision support
BCLC	ECOG performance score; number and diameter; vascular invasion and metastasis; Child-Pugh score; Okuda score	A (early stage), B (intermediate stage), C (advanced stage), D (terminal stage)	Links stage to treatment strategy
STATE	CRP; Up-to-seven criteria (tumor burden); serum albumin	Low risk >= 18 points, high risk < 18 points	Identifies patients suitable for first TACE; prognosis after initial treatment
mSNACOR	Tumor size; tumor number; baseline alpha-feto-protein; Child-Pugh; radiological response	Low-risk, interim-risk high risk	Predicts prognosis after repeat TACE, supports the decision to continue vs stop

Future directions and research gaps 

Advancements in artificial intelligence (AI), radiomics, and immunologic profiling are reshaping HCC management by enabling more personalised, data-driven approaches [3,89]. While traditional TACE selection relies on imaging and serum markers, outcome variability highlights the need for better predictive models. Radiomics and deep learning are increasingly used to analyse pre- and post-treatment imaging to forecast treatment outcomes [13,34]. A recent phase II study demonstrated that radiomic features of pre-treatment imaging could effectively predict the efficacy of TACE combined with PD-1 inhibitors, emphasising AI’s growing role in tailoring cancer therapies [90].

The integration of immunotherapy with loco-regional treatments like TACE is also gaining traction, with trials evaluating combinations such as TACE plus checkpoint inhibitors (e.g., pembrolizumab, nivolumab) and antiangiogenic agents like lenvatinib [87,91]. Additionally, accessible inflammatory markers, including NLR and CRP-to-albumin ratios, show promise in predicting treatment response and patient prognosis [34]. As clinical research evolves, a multidisciplinary approach using combination therapies based on tumour and patient-specific factors will likely become standard practice in managing the complex and heterogeneous nature of HCC [3]. Prospective multi-centre trials are necessary to collect more data on these innovative strategies.

## Conclusions

TACE remains central to managing intermediate-stage HCC, providing effective locoregional tumor control and serving as a bridge to liver transplantation. Advances in drug delivery, imaging, and adjunctive therapies have significantly broadened its clinical utility. Combination regimens involving agents such as sorafenib, lenvatinib, apatinib, and immunotherapies like atezolizumab-bevacizumab have demonstrated improved progression-free survival, though optimal combinations require further validation in large-scale trials. Emerging applications of radiomics, artificial intelligence, and inflammatory biomarkers underscore the move toward individualized, data-driven treatment approaches. Despite its overall safety, TACE carries risks, particularly post-embolization syndrome and organ-specific ischemia, necessitating meticulous patient selection and peri-procedural care. Future research should focus on refining therapeutic algorithms, validating prognostic models, and enhancing long-term outcome assessment.
